# Factors associated with overweight or obesity among post-partum women in the Tamale Metropolis, Northern Ghana: a cross-sectional study

**DOI:** 10.11604/pamj.2022.41.238.33359

**Published:** 2022-03-23

**Authors:** Hammond Yaw Addae, Rafatu Tahiru, Fusta Azupogo

**Affiliations:** 1 Nursing and Midwifery Training College, Kpembe, Box SL 98, Salaga, Ghana,; 2 Community Health Nursing Training College, Box TL 233, Tamale, Ghana,; 3 Department of Family and Consumer Sciences, Faculty of Agriculture, Food and Consumer Sciences University for Development Studies, Box TL 1882, Tamale, Ghana

**Keywords:** Post-partum women, overweight and obesity, breastfeeding, dietary intake, medical and obstetric history

## Abstract

**Introduction:**

adult overweight and obesity are public health challenges that are presently overwhelming health systems. Mothers are at an increased risk of overweight and obesity and its accompanying morbidities, especially after several deliveries; however, there is a paucity of data on the factors influencing this. As such, this study aimed to assess the prevalence and determinants of overweight or obesity among post-partum mothers.

**Methods:**

using a facility-based cross-sectional study design, mothers were selected as respondents by systematic random sampling between March and June 2018. Mothers of children less than 6 months or older than 24 months and mothers who did not attend antenatal care services were excluded from this survey. The outcome variable was overweight or obesity defined as Body Mass Index ≥ 25 kg/m^2^ and multivariable logistic regression was used to assess factors independently associated with overweight or obesity. Data was entered into and analysed using SPSS version 22.

**Results:**

analysis of 455 mothers showed that their average age was 28.0 ± 5.8 years. The prevalence of overweight or obesity was 41.8% (95% C.I = 37.2 - 46.3) and Christian mothers were twice more likely to be overweight or obese compared to their Muslim peers. Mothers who had a caesarean delivery were 36% (AOR = 1.36; 95% C.I = 1.11 - 1.66) more likely to be overweight or obese compared to those who had vaginal delivery. Mothers who consumed fresh fruits and vegetables were 42% (0.58; 0.46 - 0.72) less likely to be overweight or obese as compared to those who did not. We found a significant interaction between increasing age and parity whereby, increasing age among multiparous mothers was significantly less likely to be associated with overweight or obesity (0.92; 0.87 - 0.97) compared to primiparous mothers.

**Conclusion:**

prevalence of overweight or obesity was high, and determinants included socio-demographic factors, consumption of fruits and vegetables and gynaecological factors. Hence, strategies targeting younger women at the antenatal and delivery stages of pregnancy may improve the overall health of women by reducing caesarean sections and promoting breastfeeding.

## Introduction

At the global level, adult overweight and obesity are public health challenges that are overwhelming health systems at their current growth rate. In 2016, the World Health Organization (WHO) measured its worldwide prevalence to be 39% with overweight and obesity having a prevalence of 26% and 13% respectively [1]. It is well documented that overweight and obesity are associated with non-communicable diseases such as diabetes, hypertension, cancer and other cardiovascular challenges [2,3]. Demographic changes, economic and technological advances in low and middle-income countries have resulted in significant increases in the number of obese people, women disproportionately affected [3]. This observation is due to differences in growth rates (biological) and lack of physical activity (behavioural), which makes women more predisposed relative to their male counterparts [4]. Such high prevalence was hitherto only the preserve of high-income countries [5]. For example, in Ghana, overweight and obesity increased from 29.8% in 2007 to 39.5% in 2015 with women having a greater (55%) increase than men [6].

The prevalence of overweight and obesity among the populace in Northern Ghana, where socio-economic status is relatively low is contrastingly high (32.4%) compared to other peri-urban cities within Africa [3]. According to the 2014 Ghana Demographic and Health Survey [7], the prevalence of overweight and obesity among women of reproductive age are highest in urban areas. As the most urbanised city in northern Ghana and the third-largest city in the country [8], nutrition and public health interventions targeting overweight and obesity among post-partum women in the Tamale Metropolis may curtail the increasing trend of overweight and obesity among women of reproductive age in Ghana. There is however, a paucity of data on the prevalence of overweight and obesity among post-partum women in northern Ghana. From an intergenerational perspective, obese mothers are at risk of gestational diabetes, give birth to children with higher birth weight, who are more likely to have obesity and type-2 diabetes later in life [9,10].

Although interventions during pregnancy are known to improve the nutrition and health of the mother and child [11], interventions have focused primarily on equitable distribution and quality of care during prenatal and delivery phases. Contrastingly, the medium to long-term post-partum phase has received very marginal attention [12]. The loss of weight to an acceptable level is one of the most obvious physiologic changes, with mothers losing on average, 0.55 to 0.72 kg/month for the ensuing 6 months after delivery [13]. Some mothers may however fail to lose weight or sometimes even gain weight after delivery. The added weight has an incremental effect on subsequent pregnancies and births. As the reproductive cycle continues with subsequent pregnancies and its cumulative weight gain, the risks of maternal obesity and its accompanying challenges also increase among mothers [14]; this mechanism underpins the contribution of the post-partum period to the general development of obesity among mothers.

Although little is known about the factors that influence overweight or obesity among post-partum women, especially in developing countries, a set of complex multifactorial factors including breastfeeding (BF), dietary intake, marital status and age have been documented from the sparse literature [15,16]. Unravelling the factors which account for the general increases of post-partum overweight and obesity may be significant to improving the health of reproductive-age women and their future offspring. Considering the dearth of data, this study seeks to assess the prevalence and factors associated with post-partum overweight or obesity among mothers in Northern Ghana using Tamale Metropolis as the study area.

## Methods

**Study design and duration:** a facility-based analytical cross-sectional study design was used. The study respondents were mothers of children aged 6 to 24 months, attending postnatal care services in the 6 sub-district health facilities. Mothers were selected during these postnatal services as respondents between March and June 2018.

**Study area and setting:** Tamale Metropolis is the third-largest city in Ghana. Health-wise, the Metropolis has 6 sub-district health centres. These health centres serve as points of health care delivery for public health interventions where pregnant women and mothers go for reproductive and child welfare services; there are also about 10 private and public hospitals [8]. The 6 facilities were chosen because they form part of the Community-based Health Planning Service (CHPS) programme and hence they are expected to have a high attendance of post-partum mothers [17].

**Study participants:** the inclusion criteria were mothers who gave consent, attended Antenatal Care (ANC) services, and had a child older than 6 months but younger than 24 months of age. It was also anticipated, as established by some studies, that mothers would have lost the weight they gained during pregnancy within six months after delivery [13,18]; this informed our decision to limit the sample to mothers with children from 6-24 months of age.

**Sampling:** for each of the facilities under the study, health care workers kept a register of post-partum mothers that are scheduled for each of the days of the week as part of their normal routine activities. It was based on these weekly attendance figures that the minimum sample size is obtained for each facility. This was done by taking each facility´s weekly attendance as a fraction of all facilities´ weekly attendance and multiplying this fraction by the minimum sample size required in this survey; this gave the minimum number of questionnaires that were administered in each health facility. Furthermore, respondents were subsequently selected by systematic random sampling from the Antenatal Care (ANC) attendance register of each facility. In the sample selection process, one number from 1 to 4 was randomly selected by the lottery method. Subsequently, every fourth person on the register from the number selected was chosen for interview until the estimated sample size required for the facility was met; the next person in the register replaced any selected respondent who declined participation or did not meet inclusion criteria.

**Sample size calculation:** the sample size calculation was guided by Cochran [19].


$$n=\frac{Z^{2}*p(1-p)}{E^{2}}$$

Where p = prevalence of overweight or obesity among women of reproductive age in Ghana [3] = 32.4%. E = margin of error = 5% = 0.05 Z = standard normal deviation for 95% C.I. = 1.96. Hence minimum sample size = 336

**Data collection:** a pre-tested interviewer-administered questionnaire was used to collect data from all mothers during postnatal services. Before data collection, 5% (n=23) of post-partum women were sampled for a pre-test of the questionnaire and were excluded from the survey. The pre-tested questionnaire was used to collect data from all mothers by explaining consent in their preferred language and upon acceptance, responses from the content of the questionnaire was solicited. In the anthropometric sections of the questionnaire, the weight and height of women were measured with a Seca digital weighing scale and a Seca 206 microtoise respectively to the nearest 0.1 decimal. All measurements were duplicated and averaged to reduce random instrumental error. Where there were differences of more than 0.2cm or 0.2kg in duplicate measures, the measurement was retaken for the third time and the two closest values were used. Also, data on the medical and obstetric history were collected from the maternal health record booklet.”

Dependent variable: the dependent variable was overweight or obesity defined by the Body Mass Index (BMI), calculated as


$$\text{BMI=}\frac{\text{Weight (kg)}}{\text{Height }{\text{(m)}}^{\text{2}}}$$

Overweight or obesity was defined as BMI ≥ 25kg/m^2^.

### Independent variables

**Demographic and socio-economic characteristics:** these characteristics included maternal age (as a continuous variable), religion (Muslim/Christian), education (no education, primary/JHS and SHS/above), occupation (employed/unemployed), marital status (married, not married), wealth index (low/medium /high), gender of child (male/female) and health insurance status (no/yes). We created a household wealth index and ranked the households into tertiles of wealth using the principal component analysis [20]. The wealth index ranked households based on the ownership of durable assets including TV, satellite dish, radio, refrigerator, phone, bicycle, mattress, electric fan DVD/VCD and sewing machine and as well as the material used in building the house, the power source of the household, access to toilet facilities and fuel for cooking in the household.

**Medical and obstetric history:** data from the maternal health record booklets included age of pregnancy at first antenatal service (≤3 months/ >3 months), gestational age at delivery (<38 weeks/ 38 weeks to 42 weeks / 43 weeks and above), mode of delivery (vaginal/caesarean), obstetric abnormality during pregnancy (yes/no), place of delivery (facility-based/home), adequacy of prenatal care (no/yes), childbirth weight (<2.5kg / 2.5kg to 3.9kg / 4kg or more), parity (primiparous/secundiparous/multiparous). Other obstetric history factors, collected from verbal narration included early initiation of BF (no/yes), and mother presently BF the child (no/yes).

**Dietary intake:** we assessed the post-partum mothers´ consumption of different food groups in the last 24-hours using a 7-food group indicator which included (1) cereals, tubers and roots, (2) milk and milk products, (3) organ meat, flesh meats and fish, (4) eggs, (5) legumes, nuts and seeds, (6) dark green leafy vegetables and vitamin A-rich foods and (7) fresh fruits and vegetables. The mothers were asked to mention all foods (including drinks and snacks) they consumed in and outside the home in the last 24-hours (from wake-up to wake-up) preceding the survey. They were then probed for likely forgotten foods and to give a detailed description of foods and beverages consumed, including ingredients for mixed dishes. A score of 1, otherwise 0 was assigned if the mother consumed at least one food item from a food group.

**Quality control measures:** measures such as probability sampling of respondents so that each respondent has an equal chance of being selected at the facility level was used to minimise selection bias. Six field assistants with extensive experience were recruited and trained for 4 days. The data collection tools were pre-tested and translated into the local languages, ensuring that the information collected was appropriate and accurate. On daily basis, anthropometric tools were standardized before actual data collection.

**Statistical analysis:** data was entered into and analysed with SPSS (version 22). We used Chi-square to explore the possible associations between the outcome (overweight or obesity) and categorical/dichotomous predictor variables; one-way ANOVA was used for continuous predictors. Subsequently, univariate, and multivariate logistic regression were used to analyse the magnitude and direction of associations. Variables with P-values < 0.25 [21] in the univariate analysis were further assessed in backward stepwise logistic regression models for the predictors of overweight or obesity. The final models were selected based on the log-likelihood ratio test, Wald test and P-value. A 2-sided P-value ≤ 0.05 with a 95% Confidence Interval (CI) was considered statistically significant. Wald Chi-square test was used to test for interaction. Missing data were excluded from further analysis as it did not affect the minimum sample size required in each facility. The data at the regression analysis did not have missing values and all cases were complete.

**Ethical consideration:** ethical clearance was obtained from the Tamale Teaching Hospital´s Ethical Review Board and the study protocol was approved by the same. Additionally, authorization was granted by the Ghana Health Service (Tamale Metropolitan Health Directorate) and the management of the various reproductive and child welfare centres before the commencement of data collection. Participation in the study was voluntary and informed consent was obtained from the mothers. Participants were assured of their confidentiality and only anonymous identifiers were used, and data were reported in aggregated form.

**Data availability:** the authors have made the data that supports these findings available for editorial and review purposes. Data will be made available to interested persons upon reasonable request from the corresponding author.

## Results

**Socio-economic and demographic characteristics:** a total of 502 post-partum women were assessed. However, we excluded 47 participants from the population for analysis due to incomplete or missing data; hence the population for analysis included 455 post-partum women. The number of respondents per each of the 6 facilities was 117, 84, 87, 81, 71 and 62 for Tamale Central Reproductive and Child Health Center (RCHC), Sagnarigu RCHC, Choggu RCHC, Buipiela RCHC, Taha-Kamina RCHC and Vittin RCHC, respectively. The average age of the mothers was 28.0 ± 5.8 years. There were more Muslims (94.1%) than Christians (5.9%). About half (47.7%) of respondents were from a household with high wealth index, 79.6% were employed and a third had no education (Table 1). Table 1 also shows that almost all the mothers were married and the children were on average 12.4 ± 4.7 months of age with the proportion of males similar to female children.

**Table 1:** socio-economic and demographic characteristics of the mothers with children aged 6-24 months in Tamale Metropolis (n=455)

Variable	Overall (n=455)	Not Overweight or obesity (n=265)	Overweight or obesity (n=190)	P-value
Maternal age in years (mean, ± SD)	28.0 ± 5.8	27.20 ± 6.08	29.13+-5.18	**0.001**
Child age in months (mean, ± SD)	12.4 ± 4.7	13.0 ± 4.7	11.6 ± 4.5	**0.002**
**Religion (n, %)**				**0.007**
Muslim	428(94.1)	256(59.8)	172(40.2)	
Christian	27(5.9)	9(33.3)	18(66.7)	
**Maternal Education (n, %)**				**0.046**
No Education	143(31.4)	87(60.8)	56(39.2)	
Primary & JHS	126(27.7)	82(65.1)	44(34.9)	
SHS and above	186(40.9)	96(51.6)	90(48.4)	
**Mother's Employment (n, %)**				**0.037**
Employed	362(79.6)	202(55.8)	160(44.2)	
Unemployed	93(20.4)	63(67.7)	30(32.3)	
**Marital Status (n, %)**				0.05
Currently Married	442(97.1)	254(57.5)	188(42.5)	
Not Currently Married	13(2.9)	11(84.6)	2(15.4)	
**Wealth Index (n, %)**				0.05
Low	30(6.6)	22(73.3)	8(26.7)	
Medium	208(45.7)	128(61.5)	80(38.5)	
High	217(47.7)	115(53.0)	102(47.0)	
**Gender of Child (n, %)**				0.56
Male	218(47.9)	130(59.6)	88(40.4)	
Female	237(52.1)	135(57.0)	102(43.0)	
**Health Insured (n, %)**				0.16
Yes	419(92.9)	248(59.2)	171(40.8)	
No	36(7.1)	17(47.2)	19(52.8)	

JHS= Junior High School; SHS= Senior High School

**Dietary intake of mothers:** there were no differences between overweight or obese mothers and non-overweight or obese mothers in the consumption of cereals, tubers and roots, milk and milk products, and legumes, nuts, and seeds food groups (Table 2). Table 2 also shows that, as compared to overweight or obese mothers, non-overweight or obese mothers consumed significantly more food items from organ meat, flesh meats and fish (64.3% vs 35.7%, P=0.03), dark green leafy vegetables and vitamin A rich (62.9% vs 37.1%, P=0.001), and fresh fruits and vegetables (64.2% vs 35.8%, P=0.001) food groups.

**Table 2:** dietary intake of the mothers with children aged 6-24 months in Tamale Metropolis (n=455)

Variable	Overall sample (n =455)	Not Overweight or obesity (n=265)	Overweight or obesity (n=190)	P-value
Group 1: Cereals, tubers, and roots				0.33
Yes	436(95.8)	256(58.7)	180(41.3)	
No	19(4.2)	9(47.4)	10(52.6)	
Group 2: Milk and milk products				0.55
Yes	223(49.0)	133(59.6)	90(40.4)	
No	232(51.0)	132(56.9)	100(43.1)	
Group 3: Organ meat, Flesh meats and Fish				**0.03**
Yes	182(40.0)	117(64.3)	65(35.7)	
No	273(60.0)	148(54.2)	125(45.8)	
Group 4: Eggs				0.87
Yes	20(4.4)	12(60.0)	8(40.0)	
No	435(95.6)	253(95.5)	182(95.8)	
Group 5: Legumes, nuts, and seeds				0.59
Yes	179(39.3)	107(59.8)	72(40.2)	
No	276(60.7)	158(59.6)	118(40.4)	
Group 6: Dark green leafy vegetables and vitamin A-rich foods				**0.001**
Yes	342(75.2)	215(62.9)	127(37.1)	
No	113(24.8)	50(44.2)	63(55.8)	
Group 7: Fresh fruits and vegetables				**0.001**
Yes	335(73.6)	215(64.2)	120(35.8)	
No	120(26.4)	50(41.7)	70(58.3)	

Values in the table are frequencies (percentages)

**Medical and obstetric history:** about 6 in 10 (62.9%) of the children were breastfed within the first hour after delivery with almost all (97%) mothers still BF at the time of data collection (Table 3). Table 3 also shows that the proportion of overweight or obese mothers who had vaginal delivery was about half of their non-overweight or obese colleagues (33.5% vs 66.5%; P<0.0001). Furthermore, about a third of the mothers (29.5%) were primiparous and the proportion of non-overweight or obese mothers who were primiparous was about twice compared to their overweight or obese peers (67.2% vs 32.8%; P=0.04).

**Table 3:** medical and obstetric history of the post-partum women in Tamale Metropolis (n=455)

Variable	Frequency (n=455)	Not Overweight or obesity (n=265)	Overweight or obesity (n=190)	P-value
**Gestational age in weeks (n, %)**				**0.02**
Preterm (>38weeks)	72(15.8)	45(62.5)	27(37.5)	
Term (38 to 42weeks)	365(80.2)	215(58.9)	150(41.1)	
Post term (43weeks+)	18(4.0)	5(27.8)	13(72.2)	
**Mode of delivery**				**< 0.001**
Vaginal Delivery	245(53.8)	163(66.5)	82(33.5)	
Caesarean delivery	210(46.2)	102(48.6)	108(51.4)	
**Obstetric abnormality (during pregnancy)**				0.34
Yes	106(23.3)	66(62.3)	40(37.7)	
No	349(76.7)	199(57.0)	150(43.0)	
**Place of delivery**				**0.008**
Facility-Based	427(93.8)	242(56.7)	185(43.3)	
Home	28(6.2)	23(82.1)	5(17.9)	
**Timeliness of ANC (< =3months)**				0.28
Yes	323(71.0)	183(56.7)	140(43.3)	
No	132(29.0)	82(62.1)	50(37.9)	
**Adequacy of prenatal care (within 3 months of pregnancy and ≥ 4 times frequency)**				0.21
Yes	321(70.5)	181(56.4)	140(43.6)	
No	134(29.5)	84(62.7)	50(37.3)	
**Early initiation of breastfeeding (within 1 hour after delivery)**				**0.005**
Yes	286(62.9)	181(63.3)	105(36.7)	
No	169(37.1)	84(49.7)	85(50.3)	
**Childbirth weight**				0.09
Less than 2.5kg	76(16.7)	41(53.9)	35(46.1)	
2.5kg to 3.99kg	362(79.6)	218(60.2)	144(39.8)	
4kg and more	17(3.7)	6(35.3)	11(64.7)	
**Currently Breastfeeding**				0.36
Yes	440(96.7)	258(58.6)	182(41.4)	
No	15(3.3)	7(46.7)	8(53.3)	
**Parity**				**0.04**
Primiparous	134(29.5)	90(67.2)	44(32.8)	
Secundiparous	130(28.6)	72(55.4)	58(44.6)	
Multiparous	191(42.0)	103(53.9)	88(46.1)	

**Prevalence of malnutrition:** the mean BMI of the mothers was 24.6 ± 4.4kg/m^2^. Also, the number of underweight, normal weight, overweight and obese mothers was 21 (4.6%), 244 (53.6%), 136 (29.9%), and 54 (11.9%) respectively. Overall, the prevalence of overweight or obesity was 41.8% (95% C.I = 37.2 - 46.3).

**Multivariate determinants of overweight and obesity:** our analysis showed that post-partum women who were Christians were about twice likely to be overweight or obese compared to their Muslim peers (AOR = 1.75; 95% CI = 1.11 - 2.75) (Table 4). Further, compared to post-partum women who were married, unmarried women were about 64% (0.36; 0.15 - 0.86) less likely to be overweight or obese. The results also showed that mothers who had caesarean delivery were 36% (1.36; 1.11 - 1.66) more likely to be overweight or obese compared to those who had vaginal delivery. Additionally, mothers who consumed fresh fruits and vegetables were 42% (0.58; 0.46 - 0.72) less likely to be overweight or obese compared to those who did not. Also, the analysis found a significant interaction between increasing age and parity whereby, increasing age among multiparous mothers was significantly less likely to be associated with overweight or obesity (0.92; 0.87, 0.97) compared to primiparous mothers.

**Table 4:** multivariate logistic regression of factors associated with overweight or obesity among post-partum women in Tamale Metropolis

Variable	AOR	95% Confidence Interval	P-value
**Maternal Age (years)**	1.08	1.03, 1.13	**< 0.001**
**Religion**			
Muslim	Ref.		
Christian	1.75	1.11, 2.75	**0.02**
**Marital Status**			
Currently Married	Ref.		
Not Currently Married	0.36	0.15, 0.86	**0.02**
**Mode of Delivery**			
Vaginal delivery	Ref.		
Caesarean delivery	1.36	1.11, 1.66	**0.003**
**Group 7: Fresh fruits and vegetables**			
No	Ref.		
Yes	0.58	0.46, 0.72	**< 0.001**
**Parity**			**0.007**
Primiparous	Ref.		
Secundiparous	0.39	0.06, 2.41	0.31
Multiparous	12.00	2.54, 56.75	**0.002**
**Maternal Age x Parity**			
Maternal Age x Primiparous	Ref.		
Maternal Age x Secundiparous	1.03	0.97, 1.10	0.34
Maternal Age x Multiparous	0.92	0.87, 0.97	**0.004**

AOR, Adjusted odds ratio; Ref. = Reference category

## Discussion

This study assessed the prevalence and determinants of overweight or obesity among post-partum mothers in the Tamale Metropolis, Northern Ghana. Our findings suggest that about one-third of the post-partum women were overweight with a little over a tenth obese. To the best of our knowledge, only one study examined the prevalence of overweight or obesity among post-partum women in Northern Ghana, in which the prevalence was 38.4%; but this study was limited to post-partum women with children younger than 40 days of age [22]; hence a direct comparison of our findings with other studies is difficult because of differences in study design. Nonetheless, a systematic review covering all the regions of Ghana [3] reported an overweight or obesity prevalence of 42.5% among women of reproductive age, which is similar to our finding. The prevalence of overweight or obesity in our study was also similar to that reported in the 2014 Ghana Demographic and Health Survey (40.1%) for women of reproductive age in Ghana [23]; however, the regional prevalence for reproductive-age women (12.4%) was lower compared to our results [23]. Overall, overweight and obesity among reproductive-age women are increasing in low and middle-income countries, partly as a result of the nutrition transition and increasing sedentary lifestyles, especially in urban settings [24,25].

This study found religion, marital status, mode of delivery, consumption of fruits and vegetables, and increasing age among multiparous women as determinants of overweight or obesity. However, contrary to the literature [25,26], no significant association was observed between overweight or obesity and high household wealth as well as a high level of education. The significant association between multiparity and increasing age and their interaction effect on overweight or obesity is well established in the literature [27,28]. Parity-induced overweight and obesity could be due to pregnancy-related weight gain [29]; excessive weight accumulated during this period could increase post-partum weight retention, making shedding of weight during this phase difficult. In the wake of a reduced likelihood of initiating and sustaining BF, repeated pregnancies and deliveries could increase the odds of overweight and obesity in post-partum mothers. Cycles of cumulative excess pregnancy-induced weight gain, coupled with post-partum weight retention, could be the pathway through which parity and obesity are linked. The reduced odds of overweight or obesity with increasing age among multiparous women is partly attributed to higher adherence to BF [30] among older multiparous mothers. One implication of our finding is that BF interventions should target younger mothers and encourage the formation of mother-to-mother support groups where older multiparous mothers can transfer the requisite BF practices to younger ones.

Several studies have documented how obesity among women can affect the type of delivery and how delivery type, can, in turn, enhance the development of post-partum overweight. The latter was true in this study and has been attributed to a non-adherence to best BF practices after delivery among overweight or obese mothers [31]. Several studies indicate that Mothers who had caesarean delivery are less likely to timely initiate and sustain BF [31,32]. However, early initiation of BF and exclusive BF increase maternal energy expenditure [33] and help reduce overweight; this partly explains that mothers who had a caesarean delivery were more likely to be overweight or obese.

Additionally, the protective functions of consuming fruits and vegetables on overweight and obesity as observed in the general population are not different from post-partum women as found by this study and other studies [34,35]. Fresh fruits and vegetables contain antioxidants that neutralize free radicals and prevent their oxidative effects on other cells [36]; this, reduces stress and subsequently abnormal eating habits such as binge eating [34]. Consuming fresh fruits and vegetables with diet increases quantity but reduces the overall energy density of those diets [35]. As such, one can reach satiety without increasing energy intake, this can significantly reduce the risk of overweight and obesity. Lower energy density and increased satiety associated with fruits and vegetables are due to their high fibre (that is not digestible by humans) and water (that does not contain energy) content. This explains why post-partum mothers who consumed fresh fruits and vegetables were significantly less likely to be overweight or obese in our study. Overall, our findings suggest that intervention programmes that improve access to fruits and vegetables and promote behaviour change regarding improved consumption of fruits and vegetables among post-partum women may be beneficial in the reduction of overweight and obesity among reproductive-age women in Ghana.

The novelty with this current study is its measurement of determinants of overweight or obesity among women with children 6 to 24 months irrespective of when weight was gained. While several articles have focused on weight retention [37,38] these studies failed to account for the potential influence of excess weight by earlier pregnancies.

Equally interesting is the role religion plays in obesity, as found in this study, where Muslim mothers were less likely to be overweight or obese relative to their Christian counterparts. Though several studies report the lack of association between obesity and religion [39,40], religious inclinations play varying roles in overall health [41]. In some studies, where this association exists, an inverse relationship is often reported. For example, in a nationwide study in Mali involving 5198 women, Muslims were rather more likely to be overweight or obese [42]. There are 2 pathways that explain this association. One school of thought is about food intake restrictions in the form of fasting [41] and another is the religious prohibition of certain foods [41]. These two hypotheses are rife among Muslims than Christians [43]. But in this present study, almost all mothers were still BF at the time of data collection and BF mothers are exempted from fasting; thus, religious prohibition of certain foods such as pork [41,43] and alcohol [41] which is strictly prohibited for Muslims could be the main explanation for this association.

**Limitations of the study:** the main limitation is the inability to establish causality, occasioned by the inherent limitations of cross-sectional studies; our findings are therefore limited to the description of observed associations. The results are limited by residual confounding from measured and unmeasured confounders such as physical activity. Also, this study was conducted in health facilities as participants attending these clinics might be systematically different from those in the community; this, therefore, limits the generalizability of results in this study. Nonetheless, the bigger sample size remains a strength of this study.

## Conclusion

Our study showed that at least a third of post-partum women in the Tamale Metropolis of Ghana are overweight and about a tenth are obese. Overall, our findings suggest that intervention programmes that improve access to fruits and vegetables and promote behaviour change regarding improved consumption of fruits and vegetables among post-partum women may be beneficial in the reduction of overweight and obesity among reproductive-age women in Ghana. Overall, our findings also possibly suggest that innovative ANC services such as community-based outreach programmes targeting younger women to encourage consumption of fruits and vegetables, and reduce caesarean delivery may reduce overweight and obesity among post-partum women in Ghana. Another implication of our findings is that BF interventions from Ghana Health Service should intensify the formation of mother-to-mother support groups where older multiparous mothers can transfer the requisite BF practices to younger ones.

### What is known about this topic

- *Overweight or obesity reported in the 2014 Ghana Demographic and Health Survey was 40.1% for women of reproductive age in Ghana. This increased to 42.5% in 2016. But that among post-partum women is not known in Ghana;*
- *The higher the age of the mother, the higher the risks of being overweight or obese;*
- *Increasing wealth and education can contribute positively to overweight or obesity*.

### What this study adds

- *The prevalence of overweight or obesity among post-partum women is 41.8% and this was similar to that of women of reproductive age in Ghana;*
- *The study found that increasing age was significantly less likely to be associated with overweight or obesity in multiparity, which can be attributed to higher adherence to BF among older multiparous mothers;*
- Religion and type of delivery can have an effect on maternal overweight or obesity among post-partum mothers.
